# Muscle Synergies in Children Walking and Running on a Treadmill

**DOI:** 10.3389/fnhum.2021.637157

**Published:** 2021-05-10

**Authors:** Margit M. Bach, Andreas Daffertshofer, Nadia Dominici

**Affiliations:** Department of Human Movement Sciences, Faculty of Behavioural and Movement Sciences, Amsterdam Movement Sciences & Institute for Brain and Behavior Amsterdam, Vrije Universiteit Amsterdam, Amsterdam, Netherlands

**Keywords:** children, locomotion, development, muscle synergies, treadmill, running

## Abstract

Muscle synergies reflect the presence of a common neural input to multiple muscles. Steering small sets of synergies is commonly believed to simplify the control of complex motor tasks like walking and running. When these locomotor patterns emerge, it is likely that synergies emerge as well. We hence hypothesized that in children learning to run the number of accompanying synergies increases and that some of the synergies’ activities display a temporal shift related to a reduced stance phase as observed in adults. We investigated the development of locomotion in 23 children aged 2–9 years of age and compared them with seven young adults. Muscle activity of 15 bilateral leg, trunk, and arm muscles, ground reaction forces, and kinematics were recorded during comfortable treadmill walking and running, followed by a muscle synergy analysis. We found that toddlers (2–3.5 years) and preschoolers (3.5–6.5 years) utilize a “walk-run strategy” when learning to run: they managed the fastest speeds on the treadmill by combining double support (DS) and flight phases (FPs). In particular the activity duration of the medial gastrocnemius muscle was weakly correlated with age. The number of synergies across groups and conditions needed to cover sufficient data variation ranged between four and eight. The number of synergies tended to be smaller in toddlers than it did in preschoolers and school-age children but the adults had the lowest number for both conditions. Against our expectations, the age groups did not differ significantly in the timing or duration of synergies. We believe that the increase in the number of muscle synergies in older children relates to motor learning and exploration. The ability to run with a FP is clearly associated with an increase in the number of muscle synergies.

## Introduction

Muscle synergies reflect a common neural input to multiple muscles easing the control of complex motor tasks like locomotion (Bernstein, 1967; Bizzi and Cheung, 2013). The central nervous system can activate large groups of muscles by small sets of descending neural signals at specific moments during the gait cycle (d’Avella et al., 2003; Ting and Macpherson, 2005; Bizzi and Cheung, 2013).

When children develop walking skills, the number of muscle synergies that accompany the cyclic movement of the lower extremities increases (Dominici et al., 2011). In neonates, two muscle synergies are present resembling the reflexive stepping pattern seen at birth, while in toddlers two additional are present, i.e., a total of four synergies can be observed that persist to and during adulthood (Dominici et al., 2011; Sylos-Labini et al., 2020). The shape of the synergies’ waveforms evolves from wide, sinusoidal shapes to more focal ones with shorter activation duration from toddlers, to preschoolers and adults (Dominici et al., 2011). Here, we ask whether there is similar change in the number of synergies and the shape of their waveforms during the development of running. Is it generally true that an immature locomotor pattern is represented by fewer muscle synergies and less focal activation peaks?

Running may be defined as having a flight phase (FP) in contrast to walking where there is a double support phase (DS). Infants without independent walking experience toddling on a treadmill but with body-weight support show a shift from DS to FP at speeds of around 0.75 m/s (Vasudevan et al., 2016). Children, at the age of 6–18 years can run with FP though seemingly only in about 90% of the strides (Rozumalski et al., 2015). Given the relatively rare presence of FP, one may expect that children learning to run employ a so-called *walk-run strategy*, i.e., a mixture of DS and FP.

Running in adults differs from walking in that the activation timing changes in several muscles, amplitudes increase, or activation profiles may alter all together (Cappellini et al., 2006; Ivanenko et al., 2008; Hagio et al., 2015; Yokoyama et al., 2016). Muscle synergy analysis revealed, in particular, a shift in timing that is related to the activation of the calf muscles in line with a shorter stance phase in running compared to walking (Cappellini et al., 2006). One may ask whether such a pattern is also present in children during the development and maturation of running. In fact, already without running, the peak medial gastrocnemius activity of children at the age of 7–9 years does shift to earlier in the gait cycle during walking from 45% at comfortable speeds to 25% at fast speeds (Tirosh et al., 2013). Yet, it seems that the medial gastrocnemius muscle is pivotal for the development of walking as its full-width half-maximum (FWHM) decreases with age in typically developing children aged 1–12 years (Cappellini et al., 2016). The FWHM is a measure of the duration of the peak activation and any reduction of this measure suggests an increased ability to contract the muscle. But how do all these changes relate to (the emergence of) the aforementioned, common neural input?

We sought to answer these questions by investigating the development of both walking and running in children aged 2–9 years old. Using electromyographic (EMG) signals from 15 bilateral leg, trunk, and arm muscles, we extracted muscle synergies and related their number and waveforms with the ability to run with a FP. We expected the youngest children to make use of the afore-introduced walk-run strategy. We also hypothesized that the pivotal role of the medial gastrocnemius muscle extends to the development of running and expected its FWHM to reduce with increasing age for both walking and running. If this assumption holds, this would imply a (gradual) maturation of muscle synergies toward resemblance of adult patterns by means of an increased number of synergies accompanied by a temporal shift related to a reduced stance phase. To anticipate, we failed to find support for some of these hypotheses.

## Materials and Methods

### Participants

Thirty healthy participants were included in this study (23 children aged 2–9 years old and 7 young adults; see Table 1) with exclusion of those with known developmental disease or neurological disorders. Participant groups were selected based on the ability to manage the speeds on a treadmill with FP (∼ running, see below): toddlers (range: 25.7–40.4 months), preschoolers (range: 59.0–75.0 months), school-age (range: 78.4–106.1 months), and adults (range: 22–28 years).

**TABLE 1:** Participant characteristics [Mean (SD)].

	Toddlers	Pre-schoolers	School-age	Adults
Age	35.3 (5.6) months	66.1 (6.5) months	92.9 (9.5) months	24.15 (2.5) years
Gender (m/f)	2/3	3/3	6/6	4/3
Height (cm)	96.2 (2.8)	117.2 (7.7)	130.2 (6.8)	178.7 (5.7)
Weight (kg)	15.0 (2.0)	20.8 (3.3)	25.4 (4.9)	71.3 (8.3)

Adult participants and guardians/parents of the children provided written informed consent in compliance with the Declaration of Helsinki. Ethical approval was given by The Scientific and Ethical Review Board of the Faculty of Behavioral and Movement Sciences, Vrije Universiteit Amsterdam, Netherlands (file number: VCWE-2016-149R1).

### Setup

Participants were instructed to walk or run on the treadmill (Motek Medical BV, Culemborg, Netherlands) at a comfortable speed. Each of these conditions was repeated until a minimum of 20 consecutive strides had been recorded, where possible (Oliveira et al., 2014). When more than twenty gait cycles were recorded, the middle twenty cycles were chosen for analysis.

Walking and running were practiced and comfortable speeds were first determined by starting at a slow pace that was increased in steps of 0.1 km/h until the participant reported a comfortable speed. In two instances, participants were unable/unwilling to continue after practicing and we included the data recorded during these familiarization trials for analysis (one for walking and another for running).

Children participants wore a full-body climbing harness (CAMP Bambino Full Body Harness, CAMP USA, CO, United States) modified to also have a secure attachment point on the back at all times when on the treadmill. All participants wore own shoes for the duration of the experiment.

### Data Acquisition

#### Behavior

Vertical, mediolateral, and anteroposterior ground reaction forces were sampled at 1 kHz for every trial via the two force plates in the instrumented treadmill.

Foot switches (piezo-resistive pressure sensitive sensors: Zerowire; Cometa, Bareggio, Italy) were placed on the skin on the heel and the head of the first metatarsal underneath the foot and were secured with tape; shoes and socks were placed over the foot switches. Foot switch data were sampled at 2 kHz.

Kinematic data were recorded bilaterally using an active marker system (Optotrak motion system, NDI Measurement Sciences, Ontario, Canada) and sampled at 100 Hz. Two cameras were placed diagonally behind the treadmill and one was placed diagonally in front on the right-hand side of the participant. Single markers were attached to the right head of 5^th^ metatarsal, right lateral malleolus (LM), right lateral femoral epicondyle (LE), and right greater trochanter (GT), right and left calcaneus, right and left glenohumeral joint, right and left lateral humeral epicondyle, and right and left ulnar styloid. Here, kinematic and foot switch data merely served for step detection in the case the vertical ground reaction data were unreliable.

#### Electrophysiology

Bipolar EMG signals were recorded with a wireless system (Mini wave plus, Zerowire; Cometa, Bareggio, Italy; sampled at 2 kHz after online band-pass filtering between 10 and 500 Hz) using pediatric Ag-AgCl pre-gelled EMG disk-electrodes for children (inter-electrode distance: 19 mm: DuoTrode, Myotronics, Kent, WA, United States) and pre-gelled Ag-AgCl electrodes for adults (BlueSensor H5; Ambu, Ballerup, Denmark). Skin was cleaned with alcohol and excess hair was removed prior to electrode placement on the bulk of the muscle belly parallel to the muscle fiber direction, conform SENIAM recommendations (Hermens et al., 1999).

We targeted the following 16 bilateral muscles: tibialis anterior (TA), gastrocnemius medialis (MG), biceps femoris (BF), vastus medialis oblique (VMO), rectus femoris (RF), tensor fascia latae (TFL), adductor longus, gluteus maximus (GM), erector spinae—L2 level (ES), latissimus dorsi (LD), deltoid—anterior part (AD), deltoid—posterior part (PD), trapezius—descending part (TRAP), triceps brachii (TB), biceps brachii (BB), and brachioradialis (BR). Adductor longus was, on the basis of the quality of the recorded muscle activity, excluded for all participants for further analysis leaving 15 bilateral muscles.

A single participant was recorded in a different lab using a slightly different setup. The kinematics was measured at 100 Hz using a passive marker system (Vicon Motion Systems Ltd., Oxford, United Kingdom). The reflective markers (14 mm in diameter) were placed bilaterally in the same positions as the other participants. Twelve cameras were placed around the ceiling of the room. The treadmill (Motek Medical BV, Amsterdam, Netherlands), measured only vertical ground reaction forces. The EMG protocol and equipment did not differ from the other participants.

### Data Analysis

#### Behavior

While step events were mainly detected based on the vertical ground reaction forces (*F_v_*), they were supplemented with the events detected from the heel markers and foot switches when *F_v_* data were not sufficient for the event detection. The *F_v_* were filtered with a Savitzky-Golay filter (3rd order, 121 framelength; Savitzky and Golay, 1964). Heel strike (HS) and toe-off (TO) were defined as the first sample crossing the threshold [mean (*F_v_*)/10]. First and last HS and TO were excluded for further analysis. Heel markers were used to detect step events from the kinematics (Roerdink et al., 2008). The foot switch detection was based on an on/off algorithm. Foot switch data and kinematic data were re-sampled to 1 kHz for this application. All events were visually verified. The FP and DS were determined for up to twenty strides for every participant and condition.

All behavioral data were time-normalized to the right HS. Based on HS and TO, the percentage stance and swing of each gait cycle were determined. Velocity was normalized to leg length yielding the walking Froude number (Alexander and Jayes, 1983)

$$Fr=\frac{v^{2}}{g\times l}$$

where, *v* denotes stride speed as measured by the treadmill (m/s), *g* represents the gravitational constant (9.81 ms^–2^) and *l* is the leg length (m) as the combined measured distance of thigh (GT-LE) and shank (LE-LM). Normalizing to the walking Froude number is considered suitable when comparing gait patterns at different speeds in participants of different size (Ivanenko et al., 2004a).

#### Electrophysiology

Electromyographic data were visually inspected and artifacts were removed using a custom-written burst-detection algorithm. EMG data were high-pass (2nd order bi-directional Butterworth filter at 20 Hz; De Luca et al., 2010; Willigenburg et al., 2012) and notch filtered (bi-directional stop notch filter around *k*⋅50 Hz, *k* = 1,…,10, with half-bandwidth of 0.5 Hz). Subsequently, EMG data were rectified using the modulus of the analytic signal and finally low-pass filtered (bidirectional 2nd order filter at 10 Hz) to obtain the corresponding EMG envelopes (Oliveira et al., 2016). These envelopes were time-normalized to 200 samples per gait cycle. Right-side EMG signals were normalized to the right HS and left-side EMG normalized to the left HS.

To characterize differences in the duration of EMG activity, we computed the FWHM. The FWHM was calculated as the number of samples exceeding each cycle’s half maximum, after subtracting the cycle’s minimum. We determined FWHM for each condition as the grand average within groups and across right and left side and expressed it as a percentage of the gait cycle. While we determined FWHM for every muscle per group, in view of our hypothesis we also expressed FWHM of the MG muscle as a function of age. Moreover, we estimated the phase shift τ between the walking and running mean activity patterns of the MG muscle (Ivanenko et al., 2004b) using the cross-correlation. The cross-correlation was computed as (Nelson-Wong et al., 2009):

$$R_{\alpha \beta }\left(\Delta \right)=\frac{\int \alpha \left(t\right)\times \beta \left(t+\Delta \right)dt}{\sqrt{\int \alpha^{2}\left(t\right)dt\times \int \beta^{2}\left(t\right)dt}}$$

where α and β denote the two mean-subtracted waveforms during walking and running and refers to a time lag between the two. Then, the maximum correlation peak was determined as well as its corresponding time lag τ. Positive τ values indicate a lag of the MG signal during walking relative to running. To ease interpretation, we expressed the time lag τ in percent of the gait cycle.

For the subsequent synergy analysis, the concatenated EMG envelopes [concatenation leads to higher reconstruction accuracy (RA); Oliveira et al., 2014] were amplitude normalized to the mean value for every individual muscle (Halaki and Gi, 2012; Torricelli et al., 2014; Goudriaan et al., 2018). To increase the signal-to-noise ratio for the synergy analysis, the muscle synergy analysis was performed on each participant side (Clark et al., 2010), and thus, EMG envelopes were concatenated in a (15 muscles) × (20 strides × 200 samples) matrix for every condition and side for each participant. To ease comparison of our experimental findings with the literature, we also performed the muscle synergy analysis on only the lower limb muscles (TA, MG, BF, VMO, RF, TFL, GM, and ES), which resulted in an (8 muscles) × (20 strides × 200 samples) matrix for each participant, condition, and side (see Supplementary Material 1 for details).

For dimensionality reduction we first employed a principal component analysis (PCA) on the mean-centered data (Boonstra et al., 2015). The appropriate number of muscle synergies was determined as the minimum number required to explain 80% of the variance. Then, a rank-reduced data set was reconstructed and the mean was added back. Subsequently, we employed non-negative matrix factorization (NMF) as a decomposition tool (Lee and Seung, 1999; Tresch et al., 2006; Dominici et al., 2011; Steele et al., 2015; Rabbi et al., 2020) to identify the relevant muscle synergies. Similar to PCA, NMF is an optimization method but is supplemented by the constraint that both the extracted weighting coefficients and activation waveforms are non-negative. This accounts for the constructive (non-negative) superposition of neural and muscle activations. Following the conventional NMF approach, weightings *W* and activation waveforms *C* were estimated by minimizing the Frobenius norm between (rank-reduced) envelope data *E* and the sum of synergies (*W*×*C*, i.e., weightings × waveforms):

$${∥E-\left(W\times C\right)∥}_{F}=\text{min}$$

*E* denotes the aforementioned data, i.e., it resembles an *m*×*t* matrix (*m* = 15 muscles and *t* = 20 strides × 200 samples), the weighting coefficients *W* comprise an *m*×*n* matrix (*n* = number of synergies), and *C* contains the activation waveforms (*m*×*t* matrix) (Lee and Seung, 1999). We employed a multiplicative algorithm (Berry et al., 2007, implemented in Matlab, The Mathworks, Natick, MA, United States ver. 2019b; 200 replicates, 3,000 iterations, convergence threshold 10^−6^ and termination tolerance 10^−8^) that requires an a-priori choice of the number of muscle synergies. Capitalizing on the optimized Frobenius norm, we also estimated the RA following (Zandvoort et al., 2019; Kerkman et al., 2020) that is defined as

$$RA=1-\frac{{∥E-\left(W\times C\right)∥}_{F}}{{∥E∥}_{F}}$$

In addition, we verified that the selected number of synergies adequately reconstruct the activity of each muscle by computing the RA per muscle, condition, and participant side.

The output of the NMF is (pseudo-)random for every optimization run. Hence, we ordered the outcomes by their correlation across participants. To do so, a separate NMF analysis was carried out on the grand-average of the adult data and the waveforms were arranged based on the timing of the main peaks of the activation pattern (Cappellini et al., 2006; Santuz et al., 2020). Subsequently, this serve as a “model-order” for the outputs of the NMF from all other participants which were then correlated to that model-order and ranked based on the largest Pearson correlation coefficient.

Finally, we determined the FWHM of every activation waveform for each participant side and the time lag τ between walking and running activation waveforms.

### Statistics

Descriptive statistics included the calculation of the mean and standard deviation (SD).

#### Behavior

To test for effects of *age* on FP and for effects of *age* and *condition* (levels: instructed walking and running) on DS, we used two linear regression models. Next to main effects, the second one also served to identify interactions *age* × *condition*. The significance threshold was set to α = 0.05.

Group differences in stance duration, stride duration, and Froude values were assessed using Kruskal–Wallis tests for every condition (with corresponding Bonferroni correction for multiple comparison); note that Kolmogorov-Smirnov tests revealed significant deviations from normality arguably due to small group sizes, which let us choose for non-parametric testing. Only *p*-values below 0.01 were considered significant in order to correct for the multiple corrections.

#### Electrophysiology

Along the same lines of the behavioral data, the time lag between walking and running and the FWHM of muscle activations and the waveforms of the muscle synergies were compared non-parametrically for every condition (Kruskal–Wallis tests with Bonferroni correction). Moreover, we detailed the age-dependency of the MG’s FWHM by fitting exponentially saturating functions. To quantify the corresponding goodness-of-fit we report the adjusted *R*^2^-value unless specified otherwise.

## Results

We failed to record the aimed-for minimum of 20 strides for all participants (between 14 and 20 strides were analyzed). All the children in the toddler group were assisted with handhold by either a researcher or their parent/guardian (*N* = 5). Yet, we are confident that this did not affect the level of body-weight support during locomotion as we verified the level of vertical ground reaction forces via the toddler’s body weight (range of body-weight support was 0–7%). The conditions referred to in the following are the instructed conditions.

### Behavioral Results

**FIGURE 1:**
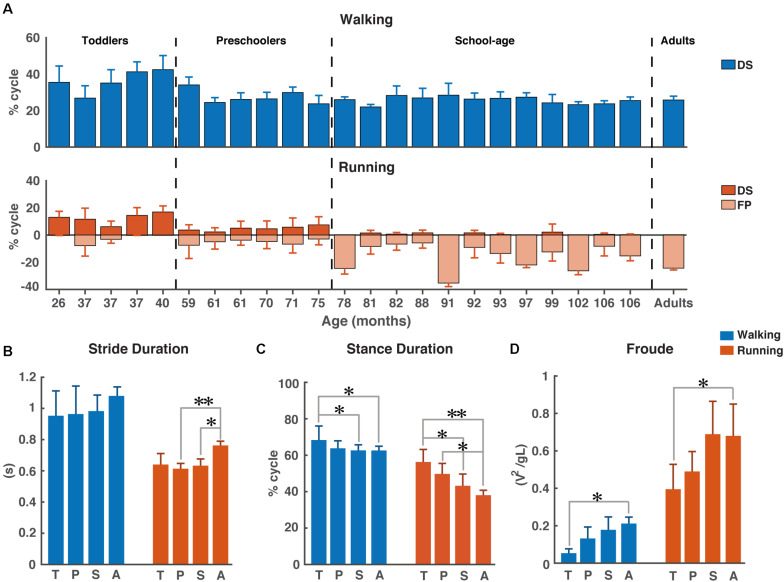
Temporal gait parameters. **(A)** Percentage double support and flight phase during walking (blue) and running (red). Flight phase is depicted with negative numbers. Vertical dotted lines separate the different groups: Toddlers, Preschoolers, School-age, and Adults, **(B)** Stride duration for walking and running, **(C)** Stance duration for walking and running, and **(D)** Froude number for walking and running. DS, double support; FP, flight phase; s, seconds; T, toddlers; P, preschoolers; S, school-age; A, adults; V, velocity, g, gravitational constant; L, leg length. *p < 0.01 and **p < 0.001.

As expected, the young children in this study used a combination of DS and FP when running on a treadmill (see Figure 1A). For FP there was a significant main effect of *age* (*p* < 0.001) and the FP increased with increasing age. A similar significance could be established for the main effect of *age* on DS (*p* < 0.001) but, opposite to FP, DS decreased with increasing age. And, there was a significant main effect of *condition* on DS (*p* < 0.001), which turned out to be smaller during running than during walking. Moreover, we found a significant *age* × *condition* interaction effect on DS (*p* = 0.0012); see Table 2 for overview.

**TABLE 2:** Linear regression estimates.

	Factor	Estimate	SE	*t*	*p*-value
**DS**	Intercept	29.59	0.39	74.95	0
	Age	−0.02	0.00	−6.31	4 × 10^–10^
	Condition running	−22.79	0.55	−41.20	<2 × 10^–16^
	Age: Condition running	−0.01	0.00	−3.25	0.0012
**FP**	Intercept	3.95	0.59	6.82	2 × 10^–11^
	Age	0.07	0.00	19.73	<2 × 10^–16^

*DS, double support phase; FP, flight phase; SE, standard error; t, t-statistic.*

We could not establish significant differences in stride duration between *groups* for walking, while during running stride duration of preschoolers and the school-age group differed significantly from that of the adults (*p* = 0.0008, *p* = 0.0061, respectively, Figure 1B). We also found a significant difference in stance duration between the toddlers and both the school-age group and the adults, both during walking (*p* = 0.0072, *p* = 0.0077, respectively) and running (*p* = 0.0045, *p* = 0.0003, respectively). And, the stance duration of preschoolers differed significantly from that in the adults for running (*p* = 0.0095); see Figure 1C.

The aforementioned differences are particularly interesting since for dimensionless speed we only found significant differences between toddlers and adults during walking (*p* = 0.0027) and between toddlers and adults during running (*p* = 0.0061); see Figure 1D.

### Electrophysiology

**FIGURE 2:**
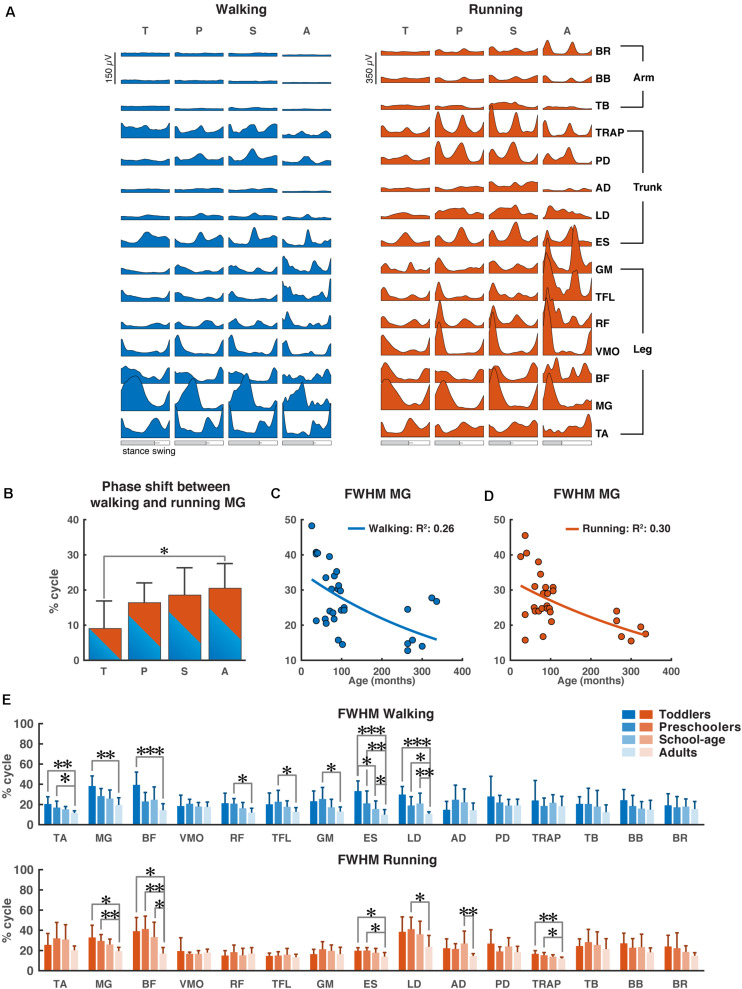
Characteristics of EMG signals. **(A)** Grand averages of 15 EMG activity patterns for walking (blue) and running (red) for all four groups, data are plotted vs. normalized gait cycle, relative duration of stance varied across groups, a bar indicates an amount of variability in the stance phase duration across groups. **(B)** Phase shift between the peak activation of medial gastrocnemius (MG) for walking and running for each group, positive value indicates a lag of walking signal relative to running signal. **(C,D)** Full-width half-maximum (FWHM) of the MG activity as a function of age for walking **(C)** and running **(D)**. Continuous lines represent exponential fittings, note the decrease in values with age. **(E)** FWHM of all muscles (means + SD) for the four groups. TA, tibialis anterior; MG, medial gastrocnemius; BF, biceps femoris; VMO, vastus medialis oblique; RF, rectus femoris; TFL, tensor fascia latae; GM, gluteus maximus; ES, erector spinae; LD, latissimus dorsi; AD, anterior deltoid; PD, posterior deltoid; TRAP, trapezius; TB, triceps brachii; BB, biceps brachii; BR, brachioradialis; T, toddlers; P, preschoolers; S, school-age; A, adults; FWHM, full-width half-maximum. *p < 0.01, **p < 0.001, and ***p < 0.0001.

The ensemble-averaged EMGs of all muscles depicted in Figure 2A appeared consistent with those reported in the literature for school-age and adult participants (e.g., Cappellini et al., 2006, 2018; Tirosh et al., 2013; Rozumalski et al., 2017).

During walking, lower leg activity had about the same overall modulation across groups with wider peaks of activity in the toddler group that was reduced in the older groups. Activity patterns in arm muscles were relatively flat during the gait cycle across all groups, while trunk muscles showed a clear modulation with increasing intensity in all the groups, but the adults. The gluteus maximus activity showed only a single major peak in the beginning of the stance phase in the toddlers, while in adults two isolated peaks were present with the additional one being early swing, in agreement with earlier reports (Olree and Vaughan, 1995; Cappellini et al., 2006, 2016; Dominici et al., 2011; Kerkman et al., 2020). Likewise, the erector spinae activity showed a single, prolonged activation peak for about 50% of the gait cycle in the toddlers, whereas in adults we could observe two distinct peaks.

During running, EMG activity increased in all muscles, but most pronounced in the adults’ lower extremities. In the toddlers, the EMG patterns of upper trunk muscles largely agreed with those of the other groups, but peak activity was less pronounced. A clear pattern of activation in arm muscles was visible in all groups except toddlers with more consistent EMG activity in the adults. The upper trunk (TRAP and PD) muscles changed from a pattern with two negligible peaks to a pattern with two prominent ones. The lower trunk muscle (ES) changed from a unimodal pattern with a small burst of activity during heel strike to a prominent bimodal pattern with bursts of activity in early stance and mid-swing (Figure 2A).

As expected, the most notable differences between the two conditions (instructed walking vs. running) were found in the time lag of peak activity of the calf muscle (MG) toward earlier in the gait cycle during running. The toddlers displayed a significantly smaller shift than the adults (*p* = 0.0071) and, when looking at all groups, there was a clear trend of shift increase with increasing age. The time lag in the toddler group had a mean (±SD) of 8.8 ± 8.0% of the gait cycle, where the others’ time lags were 16.3 ± 5.7%, 18.5 ± 7.8%, and 20.5 ± 7.1% (for preschoolers, school-age, and adults, respectively); cf. Figure 2B.

For the MG’s FWHM we found a decreasing function of age for both instructed walking and running conditions with goodness-of-fit values of *R*^2^ = 0.26 and *R*^2^ = 0.30, respectively (Figures 2C,D).

Last but not least, we found significant differences in the FWHM between groups for eight muscles in the walking condition and six muscles in the running condition (Figure 2E). In the lower leg muscles, we found significant differences between the toddler group and the adults (TA: *p* = 0.00012, MG: *p* = 0.00011, BF: *p* < 0.00006) for walking (MG: *p* = 0.0017, BF: *p* = 0.0018) and for running; between the preschoolers and the adults in the TA muscle during walking (*p* = 0.0064) and the MG and BF muscles during running (*p* = 0.0009, *p* = 0.00017, respectively); and between school-age and adults in the BF muscle during running (*p* = 0.0048). In the upper leg muscles the only differences were found in the walking condition between the preschoolers and the adults in the RF, TFL, and GM muscles (*p* = 0.0021, *p* = 0.0097, and *p* = 0.009, respectively). In the lower trunk muscles the ES muscle was significantly different between toddlers and the school-age group, toddlers and adults, the preschoolers and the adults, and finally the school-age children and the adults for walking (*p* = 0.002, *p* < 0.00001, *p* = 0.0001, *p* = 0.0085, respectively) but also the toddlers and preschoolers were significantly different from the adults (*p* = 0.0071, *p* = 0.0024, respectively) during running. The LD was significantly different between all children groups and the adults for walking (*p* < 0.00001, *p* = 0.0056, and *p* = 0.0006, respectively) and between the preschoolers and adults for running (*p* = 0.0054). In the upper trunk muscles the only differences were visible in the running condition with significant differences between the school-age group and adults in AD (*p* = 0.00026), and the toddlers as well as the preschoolers were significant different from the adults in TRAP (*p* = 0.0004, *p* = 0.0053, respectively). No significant differences were found in the arm muscles for any condition.

### Number of Synergies

**FIGURE 3:**
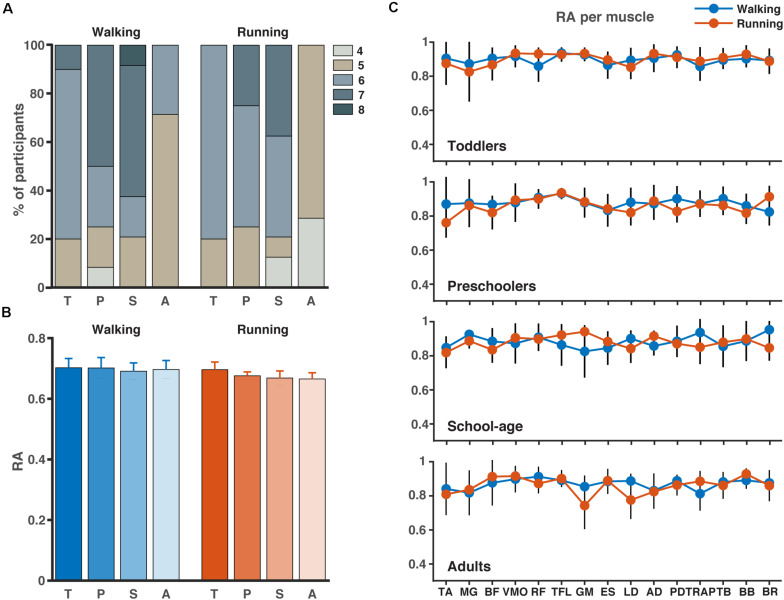
Number of synergies and accuracy. **(A)** Number of synergies needed to account for the cycle-to-cycle variability of EMG activity during walking and running for each group as determined by principal component analysis PCA (>80% of variance). **(B)** The corresponding reconstruction accuracy (RA) after rank-reduction with PCA followed by NMF. **(C)** The RA (mean SD) for each muscle and condition (blue = walking, red = running). TA, tibialis anterior; MG, medial gastrocnemius; BF, biceps femoris; VMO, vastus medialis oblique; RF, rectus femoris; TFL, tensor fascia latae; GM, gluteus maximus; ES, erector spinae; LD, latissimus dorsi; AD, anterior deltoid; PD, posterior deltoid; TRAP, trapezius; TB, triceps brachii; BB, biceps brachii; BR, brachioradialis; T, toddlers; P, preschoolers; S, school-age; A, adults.

The results for the analysis involving all muscles are illustrated in Figure 3. Across participant sides and conditions, PCA revealed that four to eight components were needed to explain 80% of the variance with the highest numbers needed for the walking condition compared to the running condition (Figure 3A). In the toddlers walking, 70% of the group required six synergies (range: five-seven), while in the preschoolers and the school-age groups, the majority required seven synergies (50% and 55%, respectively, range: four-seven and five-eight, respectively), and finally in the adult group five-six synergies were needed with 72% requiring five synergies. For running, 80% of the toddlers required six synergies (range: five-six), in the preschoolers 50% of them required six synergies (range: five-seven), and school-age group there was an almost even distribution between participants requiring six and seven synergies (40%, respectively, range: four-seven synergies), whereas in the adults five synergies explained the variance of the data for 70% of the participants, with a range of four-five. After NMF, the percentage of RA remained approximately 70% across groups and conditions (Figure 3B) and RA across single muscles exceeded 70% as a group average across conditions.

The results of the lower limb analysis and the number of synergies extracted can be found in Supplementary Material 1. Between two and five synergies were needed to explain the variance across all participants and conditions with the majority of the participants requiring four synergies during walking and the majority requiring three during running. Similarly, to the full-body analysis, the percentage RA on the lower limb analysis varied around 70%.

### Structure of Muscle Synergies

**FIGURE 4:**
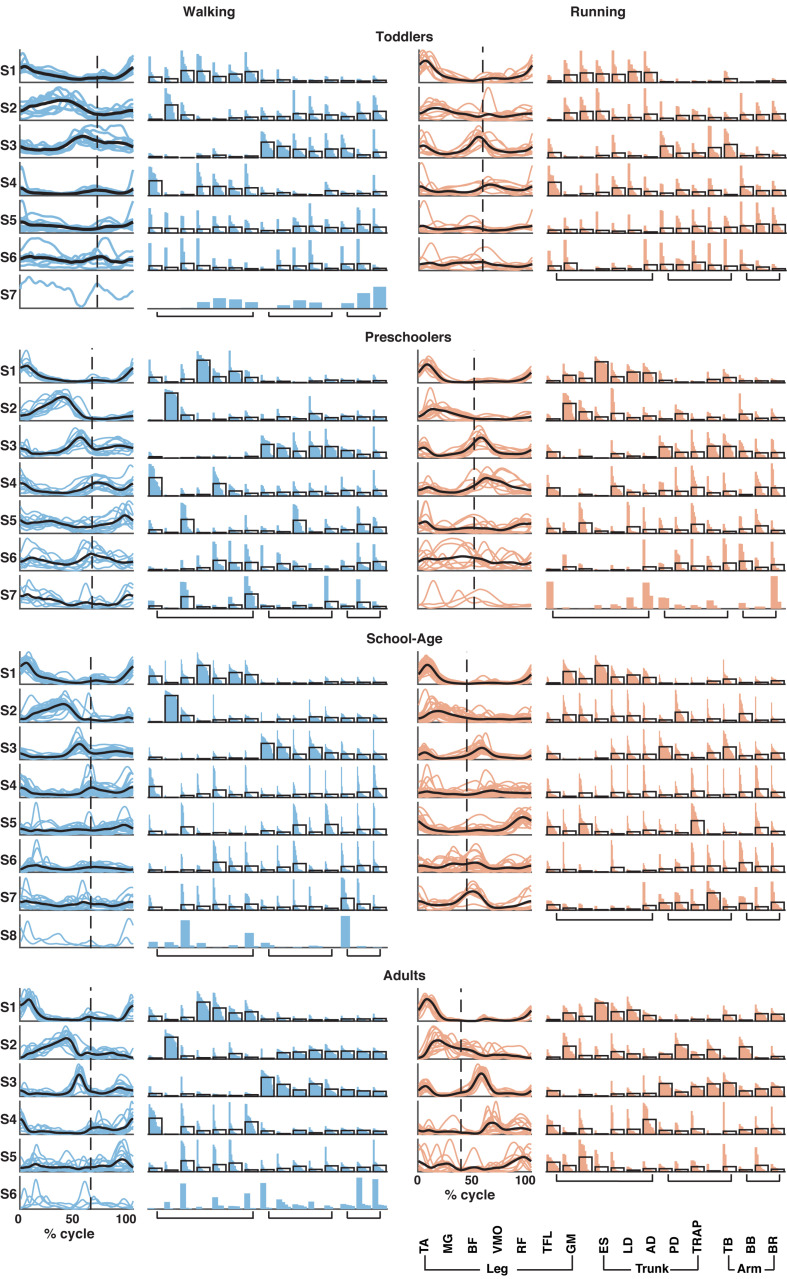
Muscle synergy structure for the four groups for walking (left; in blue) and running (right; in red). Vertical dotted line in activation timing plots represents the end of the stance phase. Each colored line represents a participant side, leading to one line for right side and one line for left side for each participant resulting in a total of (*n* = 10) for the toddler group, (*n* = 12) for the preschoolers, (*n* = 24) for the school-age group, and (*n* = 14) for the adult group. Black lines represent the mean. Y-axis is in arbitrary units. In the weighting plots, each colored bar represents the weighting coefficient for one participant side, the weightings are ordered based on their size. The black outlines represent the mean for the group. TA, tibialis anterior; MG, gastrocnemius medialis; BF, biceps femoris; VMO, vastus medialis oblique; RF, rectus femoris; TFL, tensor fascia latae; GM, gluteus maximus; ES, erector spinae; LD, latissimus dorsi; AD, anterior deltoid; PD, posterior deltoid; TRAP, trapezius; TB, triceps brachii; BB, biceps brachii; and BR, brachioradialis.

Based on the number of muscle synergies identified per participant in the previous section, the activation waveforms and corresponding weighting coefficients were grouped; cf. Figure 4. Every waveform showed a peak at a specific moment during the gait cycle. In Figure 4, the first waveform for all groups represented the loading response around the foot contact moment. On average, the lower limb muscles among others, the BF, VMO, and GM largely contributed to the first synergy during walking and running in the toddlers, while for the older children and the adults VMO contributed more to it. The second waveform peaked at mid-stance and due to the relatively shorter stance phase for running compared to walking, this pattern was shifted to earlier in the gait cycle during running compared to walking. As expected, this waveform was mostly influenced by the MG (Cappellini et al., 2006). The third waveform peaked prior to foot off in the walking condition across groups, and after foot-off for the running condition except for the toddler group, where it peaked around the foot-off event. This synergy was primarily influenced by the ES and the other trunk and arm muscles during walking and running. The fourth waveform reached its maximum at the early swing and was dominated by the TA muscle, presumably because the foot needs to clear the floor at this moment in the gait cycle, whereas the fifth waveform peaked at the end of swing in preparation for the foot contact. Higher order waveforms, if present, were more variable between participants and less defined when it comes to the main peak: the fifth synergy was not dominated by any particular muscles but predominantly influenced by the trunk and arm muscles, and this applies also to the sixth, seventh, and eighth synergies when present across groups.

**FIGURE 5:**
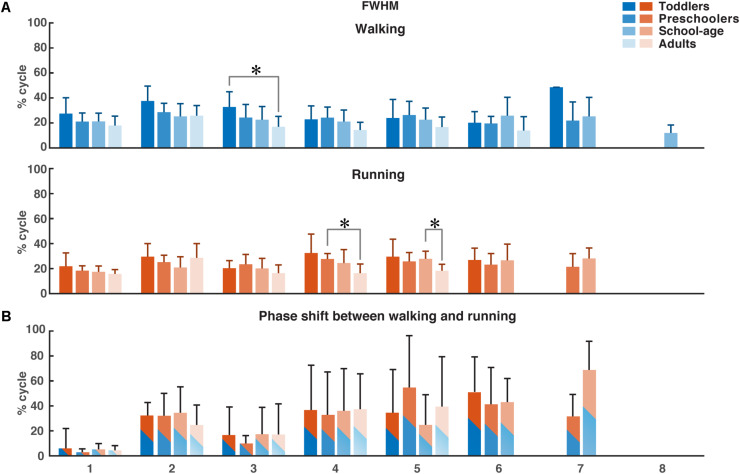
FWHM of consistent activation waveforms and phase shift between walking and running activation waveforms. **(A)** FWHM of all waveforms as a function of the percentage of the gait cycle for each group. Color-coding refer to the groups. **(B)** Phase shift as a function of the gait cycle, determined using the cross-covariance between the waveforms for walking and running. Means and standard deviations are given per group. FWHM, full-width half-maximum. *p < 0.01.

Using FWHM for the temporal activation waveforms (Figure 5), we found a significant difference between the toddler group and adult group in the third waveform in walking (*p* = 0.0013). For running, the only significant differences were found in waveform four between the preschoolers and adults (*p* = 0.0084) and in waveform five between the school-age group and the adults (*p* = 0.0074). There was a trend toward a larger FWHM in the younger groups for waveform two in walking, and a trend toward a reduction in the FWHM in running with increasing age, but with a similar duration of the FWHM in the adult group compared to the toddlers in running. There were no significant differences between groups for the phase shift of the activation waveforms between walking and running due to the large variabilities between participant sides.

Previous findings in adults from Cappellini et al. (2006) showed a characteristic time lag in the temporal activation pattern corresponding to the second synergy (weighted primarily on the calf muscles) to an early moment in the gait cycle in running compared to walking. We found a similar phase shift in all groups but no significant differences between the four groups.

## Discussion

Children make use of a walk-run strategy when learning to run. A weak exponential relationship between age and the FWHM of the MG muscle for both walking and running indicates this muscle to be important for development. We found a varying number of synergies between participant sides when investigating the muscle synergies during comfortable walking and running in 15 leg, trunk, and arm muscles in four age groups. It seems that a smaller number of synergies are active in the toddler group and adult groups compared to the preschoolers and school-age groups. Despite tendencies to wider activation patterns in the youngest groups, there were few significant differences between the groups. Yet, we did not find any significant differences in time lags between activation patterns between walking and running across the four groups.

### Behavioral Results

We found very similar stride duration and normalized speed across groups, with only few significant differences. However, we found several significant differences in the stance duration across groups in the running condition (Figure 1C). This difference in stance duration appears correlated to the split of the groups, which was based on the ability to manage the running condition with a FP; cf. Figure 1A. Here it seems that a longer stance duration with decreasing age is directly related to the reduced ability to run with a FP.

There are two traditional ways of defining running: having a FP or the kinetic and potential energies of the center of mass being in-phase. Here, we argue that all children were running despite the lack of a FP. That is, they did not have a FP in the instructed running conditions, but their double support phases differ from the double support phases observed during walking (see, e.g., Table 2). Hence, we refer to this as making use of a “walk-run strategy.” Our previous research into the development of mature running patterns revealed that even in young children walking and running are distinguishable from each other and that a multitude of kinetic and kinematic parameters can serve to discriminate between immature and mature gait patterns (Bach et al., 2021).

### Muscle Activity

Tirosh et al. (2013) found that the difference of the peak MG activation for children aged 7–9 years old was around 20% of the gait cycle between walking at comfortable and fast speeds. In this study we found a shift of around 9% of the gait cycle for the toddlers (2–3.5 years), increasing to around 16% for preschoolers (3.5–6.5 years) and 19% for school-age (6.5–9 years). Put differently, the shift between walking and running in our oldest children group was comparable to what Tirosh et al. (2013) found in their study between walking and fast walking. The fast walking speed in the study of Tirosh et al. (2013) were of similar speed as the comfortable running speeds in this study for the oldest children (0.65–0.75 Froude vs. 0.75 Froude in our study).

To test the hypothesis of the existence of a walk-run strategy, we examined the EMG patterns in the children for four types of locomotion: prescribed running with only FP, prescribed running with only DS, prescribed running with a mix of FP and DS within the gait cycle, and prescribed walking (see Supplementary Material 2). We found that the EMG patterns corresponding to the prescribed running condition are more similar to each other despite the lack of FP in terms of amplitude and pattern compared to the EMG patterns of the walking condition.

### Number of Synergies

When employing the NMF algorithm, certain *post hoc* decisions have to be made, the most important being the number of synergies to run the NMF algorithm over. The most common methods to determine this number is to either apply a threshold or to calculate the “best-linear-fit” (e.g., Cheung et al., 2005; d’Avella et al., 2006). The thresholds are applied to the centered *R*^2^-value (e.g., Delis et al., 2014; Oliveira et al., 2016; Singh et al., 2018; Booth et al., 2019; Santuz et al., 2020; Short et al., 2020), the uncentered *R*^2^-value (e.g., Torres-Oviedo et al., 2006; Kim et al., 2018; Steele et al., 2019), and the RA based on Frobenius norm (Zandvoort et al., 2019; Kerkman et al., 2020). Here, we opted for a different approach in that we first applied a PCA algorithm to the data as the outcome of the PCA is more likely to converge. After applying the PCA with a set threshold of 80% of the variance of the data explained, the data was reconstructed and after this, the NMF algorithm was applied. One may argue that continuing with the PCA rank-reduced data set would be sufficient for a muscle synergy analysis. Following this route, however, may hamper the physiological interpretation of the outcome due to negative weightings and the interpretation of them. When applying NMF, the outcome is constrained to be positive which corresponds to the summation of muscle contractions which by hypothesis are always positive. We confirmed that applying PCA followed by NMF did not greatly influence the amount of signal content lost and as such is a sound approach for the determination of muscle synergies during locomotion tasks.

We hypothesized the muscle synergies for running to “gradually” mature by means of an increased number of synergies. Our data, however, did not reveal this. Instead, we found an increase in the number of synergies with age but with a much larger number of synergies across children groups compared to the adults with a relatively larger number of synergies in the older children compared to the youngest children. Combined with what is known from motor learning and the variability we maintain in the data by concatenating across strides, the large range of synergies needed across groups and conditions to explain the variance of the data seems related to motor learning and optimizing the locomotion pattern. This is also visible in the relatively larger percentage of participant sides in the preschoolers and school-age groups needing more than six synergies in the walking condition and in the same two groups in the running condition. The relatively larger number of synergies required to explain the same variation could be due to exploration and motor learning where the lack of this increase in the toddler group could be due to the use of a “simpler” locomotor strategy to manage the tasks (Dominici et al., 2010). Adults have fine-tuned their locomotion patterns and thus we see a comparatively low number of synergies across all participant sides and conditions. The duration of the peaks of the activation patterns computed using the FWHM confirm this finding, that we consider a trend toward activation bursts for all synergies and conditions compared to the adults, who have indeed the same number of synergies as the toddlers. Another reason for the different number of synergies across groups could be due to splitting of synergies, also known as fractionization. It has been found that children aged 3–5 years, all with the ability to run over ground with a FP, show synergies that later split into more synergies for novice adult runners (Cheung et al., 2020). Likewise, the study also showed that from sedentary adults to elite adult runners, a merging of synergies occurred, which suggests that with experience, a smaller number of synergies are needed as a larger number of muscles is represented in each synergy. That is confirmed with the findings of this study. We found an increase in number of synergies from the toddlers to the school-age group, and a subsequent decrease of the number of synergies in the adult group.

### Structure of Muscle Synergies

We focused the analysis on all 15 muscles recorded from the lower limb, trunk, and upper limb, but also carried out an analysis on a subset of these muscles in order to confirm the findings from the literature where the main focus is often on the lower limb muscles (Supplementary Material 1). We found that the number of synergies across groups were much lower and comparable to what has previously been found in walking in children and adults (e.g., Dominici et al., 2011; Oliveira et al., 2016; Hinnekens et al., 2020; Mileti et al., 2020; Sylos-Labini et al., 2020) where four synergies is one of the most common findings. The activation patterns and the weighting coefficients were also comparable to what has previously been found in literature. The FWHM had smaller variability within groups which suggests that the variability we observed in the full-body analysis was due to the larger set of muscles and the possible larger contribution of the trunk and arm muscles to the waveforms.

In the synergy results of the full-body analysis there was large variability in the activation patterns and the weightings within groups. These large variabilities were participant-specific and we hypothesized that they may be related to motor-learning: children are exploring their own abilities to be able to run on a treadmill. In the adult patterns, there were a few outliers in every synergy in both activation patterns and weightings, but in general, the results were robust across participants.

There were significant differences in especially the stance duration between groups influencing the appearance of the muscle synergies. The FWHM of the synergies that appeared not significantly different between groups as a function of the full gait cycle might be considered different when identifying the relatively longer stance duration in the toddler group as running. Yet, there were fewer significant differences in the FWHM when expressed as a function of the stance duration (see Supplementary Material 3). Despite the relative differences in the stance duration for especially running, this suggests that the FWHM of the synergies did not depend on the duration of the stance phase and that differences between groups did not increase when taking the altered stance duration into account.

In the EMG signals the phase shift of the peak MG muscle activity was significantly smaller in the toddler group compared to the other groups. We expected that this would also be visible in the synergy analysis. However, we do not find any statistically significant differences in the phase shift between groups for the activation pattern (S2), commonly reported to relate to the shank muscles. In the walking conditions, the MG muscle activity clearly dominated the second synergy. In the running conditions, however, the MG muscle activity was frequently split between the first and the second synergy. This might explain why the influence of the shift in the single muscle analysis did not come to the fore in the synergy analysis.

### Limitations

One limitation in this study is the large gap in age between the participant of 40 and 59 months where, for several reasons, it was not possible to recruit and measure any children. We do not expect having this data would have changed the outcomes significantly, but it would have given a larger insight into the development of running on a treadmill in this age as well.

All children in the toddler group were assisted not only with the harness during treadmill locomotion but also with handhold from either a researcher or their parent/guardian. This did not apply to any children in any of the other groups. We verified with the recorded ground reaction forces that there were no added effects of handhold compared to the harness but the effect is present in the arm muscles on the side of the handhold as there will be less muscle activity compared to the other side. We indirectly corrected for this in the analysis by normalizing the muscle activity, not to the maximum activation for that particular muscle, but to the mean activity of that muscle. By normalizing to the mean activity of all muscles, we ensured that even muscles with low activity would not dominate the muscle synergy analysis.

Finally, all conditions referred to in this study are the prescribed conditions. This means, that the participant themselves confirmed the recorded speed was comfortable walking or running speed for them. We confirm in the Froude values that there are only significant differences between the walking speed for the toddler and adult group and the running speed between the toddler and school-age group. We also confirm that there are significant differences between the prescribed walking and running conditions for all groups (*p* = 0.0039, *p* = 0.009, *p* = 3.2⋅10^–5^, *p* = 0.0017, respectively).

### Conclusion

Children follow a walk-run strategy when learning to run on a treadmill. Older children incorporate exploratory muscle synergies when “optimizing” their walking and running pattern on the treadmill whereas the youngest children below 3.5 years of age make use of a “simpler” motor control pattern trending toward larger bursts of activation. We believe that the increase in the number of muscle synergies for individual participant sides relates to motor learning and exploration.

## Data Availability Statement

The datasets generated for this study are available on request to the corresponding author.

## Ethics Statement

The studies involving human participants were reviewed and approved by the Scientific and Ethical Review Board of the Faculty of Behavioral Movement Sciences, Vrije Universiteit Amsterdam, Netherlands (file number: VCWE-2016-149R1). Written informed consent to participate in this study was provided by the adult participants or the legal guardian/next of kin of the children participants.

## Author Contributions

MB and ND conceived and designed research. MB conducted experiments. MB, AD, and ND analyzed the data. The first draft of the manuscript was written by MB and all authors commented on previous versions of the manuscript. All authors read and approved the final manuscript.

## Conflict of Interest

The authors declare that the research was conducted in the absence of any commercial or financial relationships that could be construed as a potential conflict of interest.

## Abbreviations

AD: anterior deltoid
BB: biceps brachii
BF: biceps femoris
BR: brachioradialis
ES: erector spinae
*F*_*v*_: vertical ground reaction forces
FWHM: full-width at half-maximum
GM: gluteus maximus
GT: greater trochanter
HS: heel strike
LD: latissimus dorsi
LE: lateral epicondyle
LM: lateral malleolus
MG: medial gastrocnemius
NMF: non-negative matrix factorization
PC: principal component
PCA: principal component analysis
PD: posterior deltoid
RF: rectus femoris
TA: tibialis anterior
TB: triceps brachii
TFL: tensor fascia latae
TO: toe-off
TRAP: trapezius
VMO: vastus medialis.
