# Supplementary material for: Muscle Synergies in Children Walking and Running on a Treadmill
Source: Front Hum Neurosci. 2021 May 10;15:637157. doi: 10.3389/fnhum.2021.637157 (PMC8143190; doi:10.3389/fnhum.2021.637157)
Supplement: Supplementary file 1 [file Data_Sheet_1.pdf]

1 Supplemental Material 1

Muscle synergy analysis of the 8 lower limb muscles, tibialis anterior (TA), medial gastrocnemius (MG), biceps femoris (BF), vastus medialis (VMO), rectus femoris (RF), tensor fascia latae (TFL), gluteus maximus (GM), and erector spinae (ES). The muscle synergy analysis was carried out in a similar fashion to the main text. Briefly, a principal component analysis (PCA) was applied to the mean-centered data with a cut-off of 80% of the variance explained. The mean was then added back to the now reconstructed rank-reduced data-set. Subsequently a non-negative matrix factorization (NMF) was applied on this rank-reduced data set and run for the corresponding number of synergies for each participant side. Supplemental Figure 1A shows that on the lower limb analysis the chosen threshold lead to two-to-five synergies per group and condition. The number of synergies per participant side was larger for the walking condition compared to the running condition where more participants only required three synergies. Supplemental Figure 1B shows that a corresponding reconstruction accuracy (RA) based on the Frobenius norm of the output of the NMF on the rank-reduced data-set resulted in a range of 65-75%. Supplemental Figure 1C shows that for the number of synergies required for each participant side, result in an average of more than 75% reconstruction accuracy for each muscle.

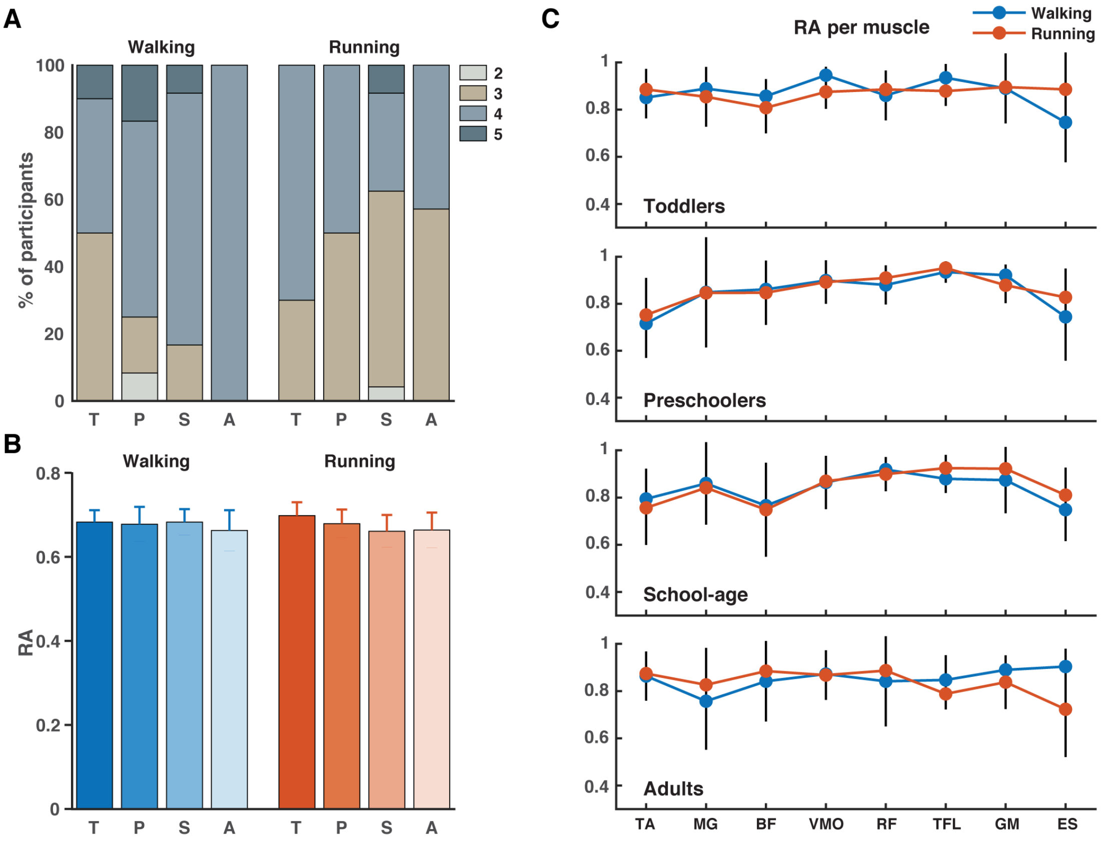

Supplemental Figure 1. Number of synergies based on lower limb analysis and accuracy of the muscle synergy analysis. A) Number of synergies needed to account for the cycle-to-cycle variability of the lower limb EMG activities during walking and running for each group as determined by principal component analysis PCA (>80% of variance). B) The corresponding reconstruction accuracy (RA) after rank-reduction with PCA followed by NMF. C) The RA (mean ± SD) for each muscle and

condition (blue = walking, red = running). Abbreviations TA, tibialis anterior; MG, medial gastrocnemius; BF, biceps femoris; VMO, vastus medialis oblique; RF, rectus femoris; TFL, tensor fascia latae; GM, gluteus maximus; ES, erector spinae; T, Toddlers; P, Preschoolers; S, School-age; A, Adults.

The activation waveforms and the weighting coefficients of the muscle synergies of the lower limb analysis can be found in Supplemental Figure 2. The waveforms of the first synergy peaked just after heel strike, ~5-10% of the gait cycle, and are mostly loaded on VMO, RF, TFL and GM, providing body support during weight acceptance. The waveform belonging to the second muscle synergy peaked around mid-stance for all groups and conditions and due to the relatively shorter stance duration for running, the peak is shifted to earlier in the gait cycle. The second synergy is mostly loaded by the MG muscle, especially in walking whereas in running also the hamstring (BF) become involved. The peak of the waveform belonging to the third synergy peaked just before foot-off in the walking condition and just after in the running condition for the older children and adults, and around the foot-off for toddlers. The muscle with the largest contribution to this muscle synergy is TA for foot lift, and ES. The waveform belonging to the fourth synergy peaked during swing and was variable in which muscles contribute the most. In the case of a fifth synergy, the same applies, with large variability between participants.

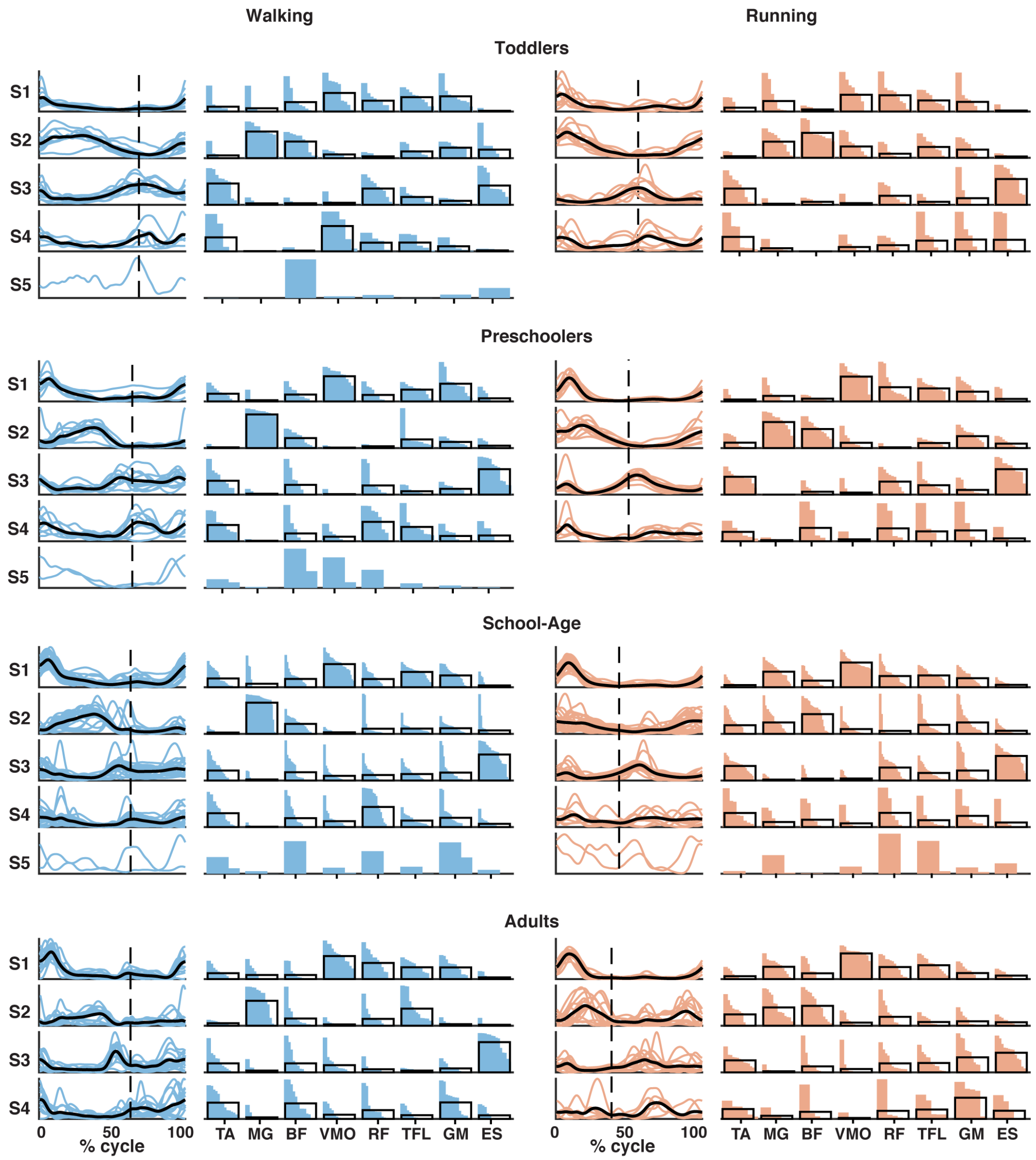

Supplemental Figure 2: Muscle synergy structure for the four groups for walking (left in blue) and running (right in red). Vertical dotted line in activation timings plots represents the end of the stance phase. Each colored line represents a participant side, leading to one line for right side and one line for left side for each participant resulting in a total of (n=10) for the toddler group, (n=12) for the preschoolers, (n=24) for the school-age group, and (n=14) for the adult group. Black lines represent the mean. Y-axis is in arbitrary units. In the weighting plots, each colored bar represents the weighting coefficient for one participant side, the weightings are ordered based on their size. The black outlines represent the mean for the group. Abbreviations: TA, tibialis anterior; MG, gastrocnemius medialis; BF, biceps femoris; VMO, vastus medialis oblique; RF, rectus femoris; TFL, tensor fascia latae; GM, gluteus maximus; ES, erector spinae.

The results of the FWHM analysis of the lower limb analysis can be found in Supplemental Figure 3. We found significant differences in the second synergy in the activation duration, where the toddler group had a significant longer activation duration than all other three groups (p = 0.0038, p = 0.00051,

$p = 6 \cdot 10^{-5}$, respectively). We also found a significant difference in the third synergy between the toddlers and the adults ($p = 0.0058$). Finally, there were no significant differences in the running condition, however, there was a trend towards the toddlers being significantly different from the adult group ($p = 0.0162$) in the second waveform. There were no significant differences in the phase shift between groups, similarly to the whole-body analysis.

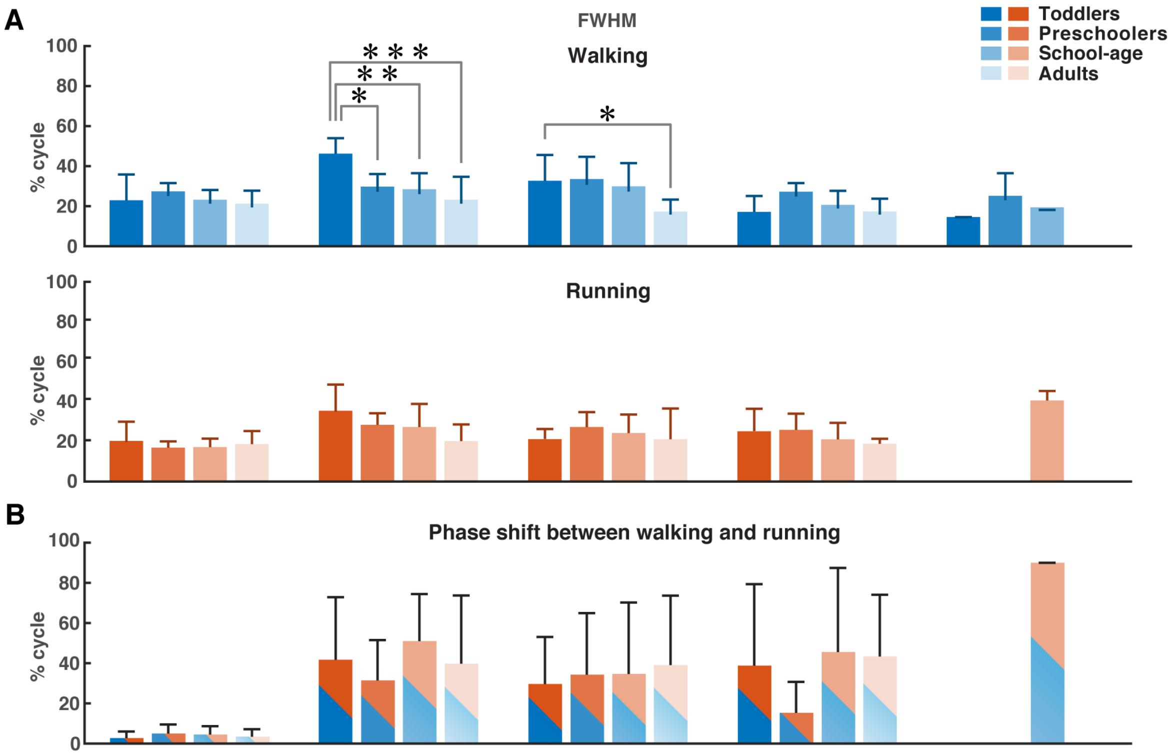

Supplemental Figure 3: FWHM of consistent activation waveforms and phase shift between walking and running activation waveforms. A) FWHM of all waveforms as a function of the percentage of the gait cycle for each group. Colour-coding refer to the groups. B) Phase shift between walking and running activation waveforms as a function of the gait cycle, determined using the cross-covariance between the waveforms for walking and running. Means and standard deviations are given per group. Abbreviations: FWHM, Full-width half-maximum. * = $p < 0.01$, ** = $p < 0.001$, *** = $p < 0.0001$.

2 Supplemental Material 2

EMG patterns for children participants split into four categories based on the presence of the DS or FP in the gait cycle: prescribed running with only FP, prescribed running with only DS, prescribed running with both FP and DS present (Mix) in the gait cycle, and prescribed walking.

We show that the walking condition is different from the prescribed running conditions independent of the behavioral patterns, i.e., flight phase or double support phase or a mix thereof.

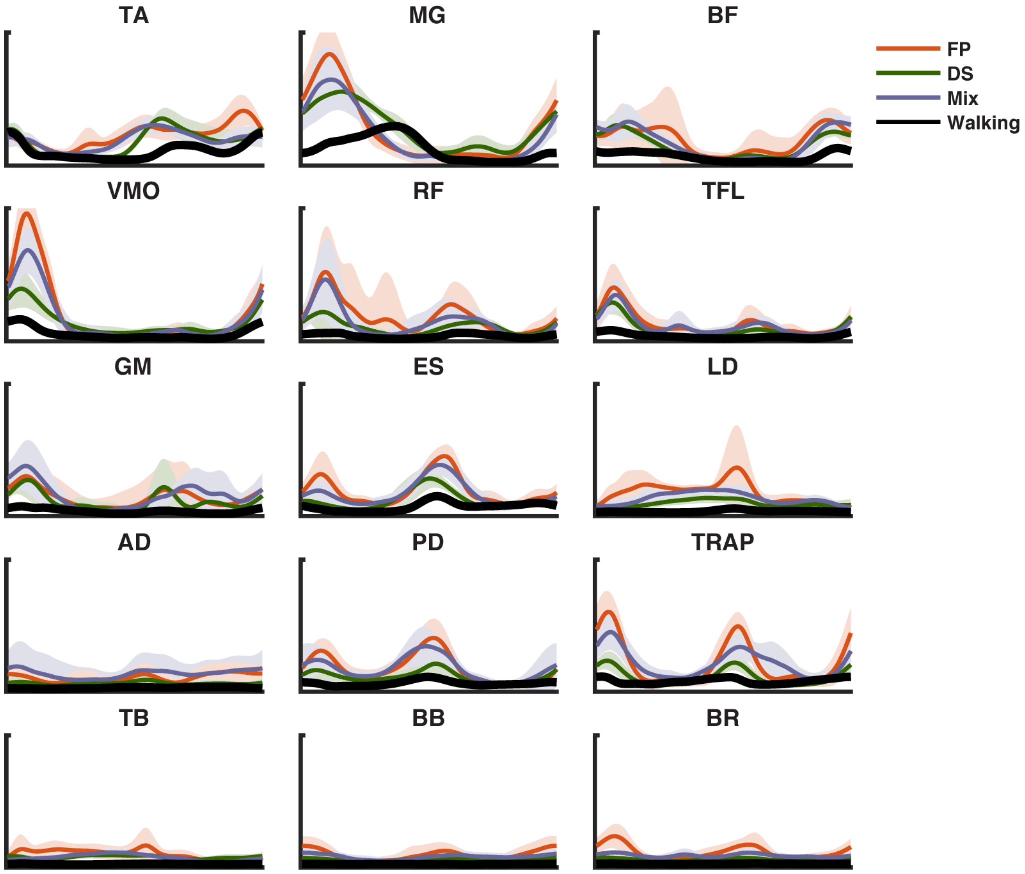

Supplemental Figure 4: Ensemble averaged EMG patterns of the children participants split into behavioral patterns. Four patterns are shown: prescribed running with only flight phase (FP in red), prescribed running with only double suppport (DS in green), prescribed running with a mixture of FP and DS within the gait cycle (Mix in blue) and prescribed walking (black). The shaded areas refer to the standard deviations across participants for the three prescribed running conditions. Abbreviations: TA, tibialis anterior; MG, gastrocnemius medialis; BF, biceps femoris; VMO, vastus medialis oblique; RF, rectus femoris; TFL, tensor fascia latae; GM, gluteus maximus; ES, erector spinae; LD, latissimus dorsi; AD, anterior deltoid; PD, posterior deltoid; TRAP, trapezius; TB, triceps brachii; BB, biceps brachii; and BR, brachioradialis.

3 Supplemental Material 3

FWHM of synergy one-eight, of the full-body analysis, expressed as a percentage of the mean stance phase for each group can be found in Supplemental Figure 5. Data are presented as the group means and error bars represent standard deviations. There is only one significant difference between the groups in terms of FWHM which are related to synergy three for walking ($p = 0.0086$). There is a trend for the FWHM for running to be larger with younger age, whereas there is a trend towards the opposite for walking. This could be a factor of the relatively longer stance phase in running for the youngest group compared to the other groups.

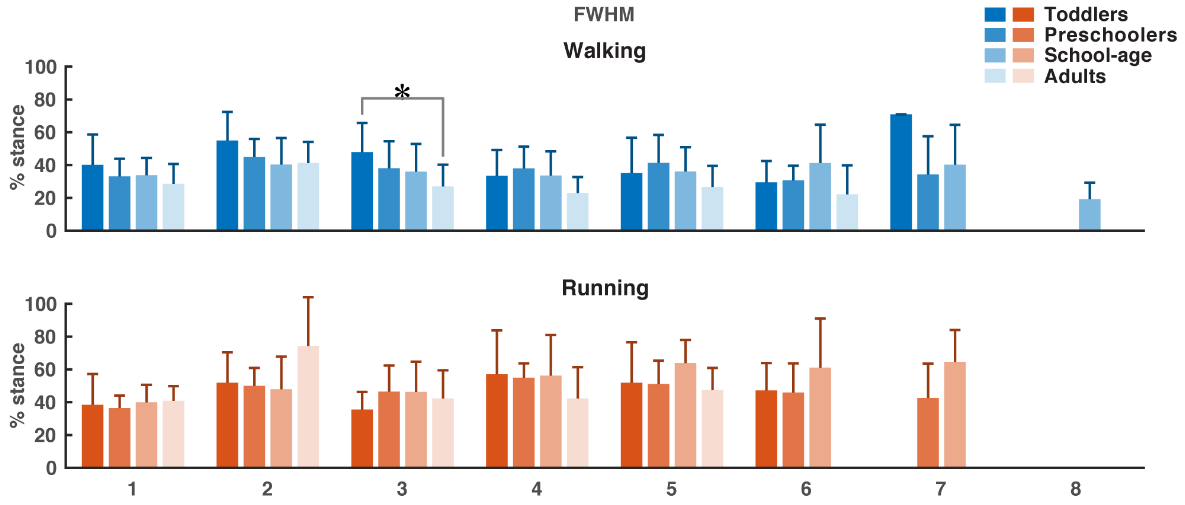

Supplemental Figure 5: FWHM of consistent activation waveforms of full-body analysis, expressed as percentage of the mean stance phase duration. Color-coding refer to the groups. Means and standard deviations are given per group. Abbreviations: FWHM, Full-width half-maximum. * = $p < 0.01$.
